# Language representation and presurgical language mapping in pediatric epilepsy: A narrative review

**Published:** 2020

**Authors:** Mahdieh Karami, Reza Nilipour, Majid Barekatain, William D Gaillard

**Affiliations:** 1 PhD of Cognitive Science of Language, ICSS, Tehran, Iran.; 2 Emeritus Professor of Neurolinguistics and Clinical Linguistics, Department of Speech Therapy, University of Social Welfare and Rehabilitation Sciences, Tehran, Iran; 3 Professor of Neuropsychiatry, Department of Psychiatry, School of Medicine, Isfahan University of Medical Sciences, Isfahan, Iran.; 4 Professor of Neurology and Pediatrics, George Washington University, Center for Neuroscience and Behavioral Health, Children’s National Medical Center, Washington DC. USA

**Keywords:** Pediatric epilepsy, language mapping, presurgical evaluation, language laterality

## Abstract

As one of the most common neurological diseases in children, epilepsy affects 0.9–2% of children. Complex interactions among the etiologies of epilepsy, interictal discharges, seizures, and antiepileptic drugs lead to cognitive impairments in children with epilepsy. Since epilepsy is considered as a network disorder, in which seizures have a widespread impact on many parts of the brain, childhood epilepsy can even affect the normal development of language. About 25% of children with epilepsy do not respond to medications; therefore, brain surgery is considered as a treatment option for some of them. Presurgical neuropsychological evaluations including language mapping are recommended to preserve cognitive and language abilities of patients after surgery. Functional magnetic resonance imaging as a non-invasive technique for presurgical language mapping has been widely recommended in many epileptic centers. The present study reviewed language representation and presurgical language mapping in children with epilepsy. Mapping language in children with epilepsy helps to localize the epileptogenic zone, and also, to predict the cognitive outcome of epilepsy surgery and possible cognitive rehabilitation. This review collected information about language representation and language mapping in pediatric epilepsy settings.

## Introduction

As a neurological disease, recurrent and unprovoked seizures are the main symptoms of epilepsy (1). Epilepsy has been classified into two broad categories: generalized and focal seizures (2). Generalized epileptic seizures are conceptualized as originating at some point within, and rapidly engaging, bilaterally distributed networks (3,4 ). On the other hand, a focal seizure starts in a distinct region (epileptogenic zone) and spreads locally to affect one part of the brain. It may become secondarily generalized to the whole brain (3 ). As one of the most common neurological diseases in children, epilepsy affects 0.9–2% of children (3). Pediatric epilepsy syndromes, organized by the age of presentation, are classified into four types including neonatal onset (including benign neonatal seizures, benign familial neonatal epilepsy, etc.), infantile onset (such as myoclonic epilepsy in infancy, benign familial infantile epilepsy, etc.), childhood (e.g., acquired epileptic aphasia [Landau-Kleffner syndrome (LKS)], benign epilepsy with centrotemporal spikes [benign rolandic epilepsy], and so on) and adolescence to adult onset (juvenile absence epilepsy and mesial temporal lobe epilepsy with hippocampal sclerosis) [for more details, see (5)].

**Cognition and language in pediatric epilepsy**

Complex interactions among the etiologies of epilepsy, interictal discharges, seizures, and antiepileptic drugs lead to cognitive impairments in epilepsy (6 Moreover, changes in genome, gene expression, receptor characteristics, and peptides along with brain injury are responsible for both seizures and functional abnormalities underlying cognitive impairment (7 ). 

**Figure:**
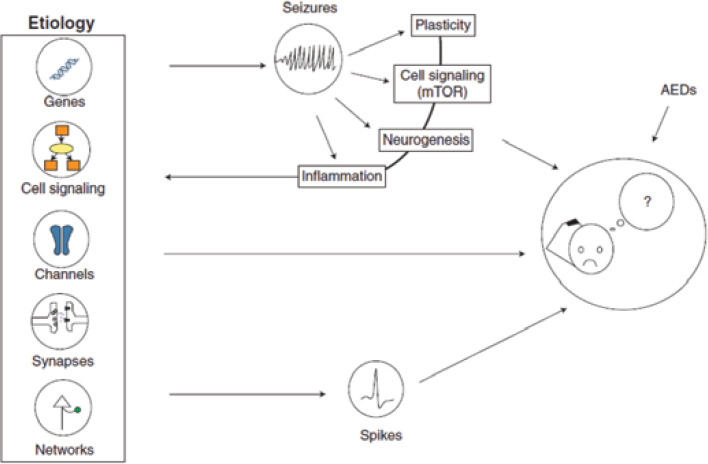
A summary illustration of the ensemble of processes leading to cognitive impairment in epilepsy. mTOR, the mammalian target of rapamycin; AEDs, antiepileptic drugs (7)

Frequent focal interictal discharges affecting the perisylvian regions, without any significant brain lesion or neurologic history, are the main characteristics of benign epilepsy of childhood with centrotemporal spikes (BECTS), which is a localization-related seizure disorder (8). About 15–25% of cases of pediatric epilepsy suffer from BECTS or Rolandic epilepsy as the most common focal epilepsy syndrome in childhood (9). Seizures stopping after puberty are considered as benign. Affected children typically have a normal full-scale IQ (10). However, some deficits are reported in neuropsychological features such as language, attention, and memory (11). The majority of educational problems in children with BECTS are attributed to language impairment in the affected children (12). 

Children’s language development studies started in the 1960s (13). It has been claimed that the same frontal-temporal network is activated in children (14). Moreover, the risk of language impairment has been reported in children with focal epilepsy (15 ). 

Since epilepsy is considered as a network disorder, and seizures have a widespread impact on many parts of the brain, childhood epilepsy can affect the normal development of language. It is not known how childhood-onset epilepsy affects functional language networks (16). Language dysfunction is caused by acute seizures or epileptiform discharges, and also occurs in chronic changes to underlying networks (17). Epileptic activity in BECTS may disturb the cerebral organization for language (18). Whether this language dysfunction is transient in nature or results in a permanent disturbance of language development is a question (19).

It is claimed that a genetic mutation or a structural lesion causes both seizures and language disorders, because even after seizure control, language problems are reported in children with new onset seizures, and are not always resolved (17). Speech and language impairments are rarely observed in children with left hemisphere focal brain injury, which is due to the plasticity of the developing brain. The related factors have been reported as to lesion location, size, and etiology, as well as age at seizure onset (20).

Overvliet et al. (21) stated that language dysfunction could lead to academic underachievement in addition to long-term psychological, social, and professional problems. It has also been claimed that children with poor academic achievement frequently have undiagnosed language difficulties (22).

Some recent studies have proposed that there are dorsal and ventral processing streams connecting Broca’s area with the temporal and parietal language cortex (23). The ventral stream is implicated in semantic processing, while the dorsal stream is involved in phonological processing, syntactic processing, and working memory (16). The dorsal auditory processing stream connects regions important for the processing of speech phonemes with regions necessary for expressive production of phonemes in posterior Broca’s area (23,24). The dorsal stream translates speech signals into articulatory representations (25). The ventral auditory processing stream links auditory input with conceptual meaning, which is represented across widely-distributed regions (the lexical-semantic system) (23). 

It appears that the ventral stream tracts mature early in infancy, while the tracts of the dorsal stream (arcuate fasciculus) continues to develop into adolescence (26). Croft et al. (16) showed decreased activity in the ventral components of the language network in children with epilepsy with a left hemispheric focus in comparison with the dorsal components. Therefore, they may show poorer language function compared to normal children. 

Language laterality (lateralization) is the phenomenon, in which one hemisphere shows greater involvement in language functions than the other (27). To determine language dominance, researchers use qualitative/subjective methods by visual rating or the quantitative laterality index (LI) (28). Gaillard et al. (29) revealed in their study that visual interpretation of language laterality was comparable to quantitative regions of interest (ROI) analysis.

Some researchers have reported that developmental aspects of language laterality in typically developing children are not fully characterized (30). Some other researchers have also reported hemispheric differences in infants from the fetal period onward ( 31, 32). Language lateralization is also reported in neonates and infants (31 ). 

There are contradictory findings about age-related language lateralization. Some studies have reported that lateralization continues to increase with age (33). On the other hand, some other studies have reported that lateralization is comparable to adults at the age six or seven (34). Vargha-Khadem et al. (35) reported that the potential for language relocalization would decrease after the age of five.

Weiss-Croft and Baldeweg (36) conducted a systematic review of 22 years of fMRI and found that changes in language lateralization were minimal after five years of age. Dehaene-Lambertz et al. (32) collected fMRI images from 20 three-month-old healthy nonsedated infants listening to speech. The results revealed activation in a large subset of the temporal lobe with a significant left-hemispheric dominance. Dehaene (31) stated that responses to the native language were more left lateralized in comparison with other vocal sounds (36). 

Berl et al. (30) investigated the degree of lateralization of fronto-temporal and modulatory prefrontal-cerebellar regions in 57 typically developing children. The results showed that temporal regions were strongly and consistently lateralized at the age of seven. However, frontal regions were less strongly lateralized at the age of ten. They also found that modulatory prefrontal-cerebellar regions were the least strongly lateralized, and that the degree of lateralization was not associated with age. 

Shtyrov et al. (37) carried out a research to observe whether language laterality is explained by physical or linguistic features of speech. They found that physical properties or the phoneme status of a sound were not sufficient for laterality; left hemispheric dominance was observed only when the sound was placed in word context. They concluded that language laterality was bound to the processing of meaningful sounds.

Imaging studies have shown atypical language laterality and the asymmetry of cortical activity during linguistic tasks in patients with epilepsy (38). Atypical language patterns may represent re-organization (the primary region of language processing has moved) or compensation (additional areas are recruited within broadly-distributed networks to assist in language processing) (20). Language in epileptic patients might be displaced to the contralateral hemisphere, or be reorganized within the same hemisphere (38). You et al. (20) found three activation patterns in typically developing and epileptic children; the typical distributed network expected for tasks in left inferior frontal gyrus and along left superior temporal gyrus; a variant on the left dominant pattern with greater activation in IFG, mesial left frontal lobe, and right cerebellum; and activation in the right counterparts of the first pattern in Broca’s area.

Other fMRI studies also showed atypical language lateralization in children with epilepsy (39, 40). Right lateralized language in children with early left-hemispheric epilepsy is much more likely compared with the general population (41). More bilateral activation for language tasks that are left-lateralized is observed in these children, including verb generation, sentence generation, and semantic decision tasks, in addition to right-lateralized tasks, such as prosody discrimination (42). About 77% of patients with epilepsy have an atypical pattern for language (right or bilateral) (43, 44). 

Gaillard et al. (38) investigated the relationship between partial epilepsy, MRI findings, and atypical language representation. They reported that early seizure onset and atypical handedness, as well as the location and nature of pathologic substrate, were important factors in language reorganization.

Some studies have also reported different findings about types of epilepsy with a larger effect on language. Other studies have also found that children with absence epilepsy have worse language performance than children with focal epilepsy (42).

Ojemann et al. (45) reported language disturbances as side effects of antiepileptic drug topiramate (TPM) and zonisamide (ZNS) therapy. TPM and ZNS both contain a sulfa moiety. It is claimed that verbal processing may be especially sensitive to sulfa-containing drugs (45). The results of some studies on speech and language disorders are summarized in Table 1 (cited in 42). 

**Table 1:** Speech and Language Disorders (45)

Auditory agnosia	Inability to recognize the symbolic meaning behind a sound, including an inability to understand speech or meaningful noises (such as a telephone ring)

Aphasia	Disorders affecting the production or comprehension of spoken and written language due to acquired damage to the language regions of the dominant (typically left) hemisphere. Different components of language are affected depending on the area of brain damage. Although the disorders described below are the canonical aphasias, patients typically have mixed symptoms.
	*Receptive/Fluent/Wernicke’s Aphasia*: Inability to understand spoken or written language, classically attributed to damage of the superior temporal gyrus of the dominant temporal lobe. Speech is fluent but nonsensical.
	*Expressive/Non-fluent/Broca’s Aphasia*: Inability to produce speech or writing, classically attributed to damage of the inferior frontal gyrus of the dominant frontal lobe. Speech is halting and grammar is significantly affected, but comprehension is typically spared.
	*Conduction Aphasia*: Inability to repeat secondary to damage to the arcuatefasciculus which connects Wernicke’s and Broca’s areas.

Dysarthria	Impairment of speech due to difficulty with strength or coordination of the muscles of speech. This can be a primary muscle problem or secondary to damage to nerves or brain structures that control the muscles. Mistakes in speech are usually consistent, and there can be difficulty in other functions like chewing or swallowing. Dysarthria can be a congenital or acquired condition.

Prosody	The varying rhythm, intensity, or frequency of speech that, when interpreted as stress or intonation, aids in transmission of meaning.
	*Aprosody*: Absence of rhythm or normal pitch variations; “robotic” voicing.
	*Dysprosody*: Impairment in normal speech intonation patterns.

Speech Dyspraxia/Apraxia	Difficulty in articulation of syllables or words due to impaired motor planning; mistakes are inconsistent, with intermixed fragments of intact speech. There is often impaired pitch and prosody. Unlike in dysarthria, muscle strength and coordination are otherwise intact. Dyspraxia can be a congenital or acquired condition.

Patients with epilepsy-aphasia spectrum, including LKS and benign epilepsy, share features of sleep-potentiated EEG abnormalities, rare or even absent clinical seizures, and cognitive problems (46).

Children with LKS, as the canonical example of the epilepsy-aphasia spectrum with previously normal development, face a progressive language regression. They may lose the ability to understand speech, and speech production diminishes in the end (42). 

**Presurgical evaluation** **in children with intractable epilepsy **

It has been reported that about 25% of children with epilepsy do not respond to medications (47). Brain surgery is considered as a treatment option for 10 to 50% of patients with intractable epilepsy (Engel, 2018). The main purpose of neurosurgery in pediatric epileptic patients is reduction of the frequency of seizures (20). The major result of this rehabilitation procedure can promote quality of life in these patients.

Epilepsy surgery is considered as highly successful if children with intractable focal epilepsy are selected carefully based on presurgical evaluations (48). It has been reported that 58–74% of carefully selected patients become seizure free, and 67–82% exhibit favorable seizure control (49). 

Téllez-Zenteno et al. (50) conducted a meta-analysis study on children undergoing brain surgery. The results indicated that after 1-year follow-up, 74% of children with brain lesions and 45% of those without lesions became seizure-free. 

Presurgical assessment including data from video electroencephalographic (video EEG) recordings, structural and functional imaging as well as neuropsychological and clinical assessments are considered necessary for presurgical structural and functional mapping to preserve cognitive and language abilities after surgery.

It has been claimed that brain anatomy alone cannot show language areas and may cause risk of injury in epilepsy surgery (45, 51). To determine the language locality of the eloquent cortex and laterality, fMRI is recommended as a non-invasive method. The sensitivity and specificity of fMRI for language lateralization are reported to be about 80 and 90%, respectively (17).

fMRI is based on the observation that increased neuronal activity is associated with tightly-regulated and regionally-specific increases in the cerebral blood flow (52). Detecting the location of blood oxygen level dependent MR signal changes induced during cognitive tasks (involving language, memory, and motor control) allows the mapping of neural networks involved in the performance of the tasks (29). Medina et al. (53) reported that fMRI helped identify brain areas related to ictal or inter-ictal activities in children with epilepsy (53).

Considering the challenges of fMRI language mapping in clinical settings, the procedure has been recommended to be applied in children with epilepsy, even as young as 5–7 years of age (54). 

The most commonly used tasks for presurgical language mapping with fMRI are based on verbal fluency to identify expressive language functions in the dominant hemisphere (29). Verbal fluency paradigms can reliably lateralize language processing in children (55). Verbal fluency requires generating words from given letters (e.g., C, L, F for children; F, A, S for adults), generating words related to specific categories (e.g., food, animals, etc.), or changing a verb to a presented noun (target, ‘‘ball’’; response, ‘‘catch, throw, pitch’’) (29). It is reported that modified forms of verbal fluency tasks can be used in children as young as five years old (56). Vakharia (57) reported that verbal fluency and verb generation could determine laterality rather than localize language functions precisely. 

Language mapping with the same or developmentally-adapted paradigms leads to similar results in children as in adults (29). Although the general principle of mapping language for children is the same as that for adults, there are many challenges in the mapping process and task design (43). 

.To the best of our knowledge, there is no documented report on pediatric language mapping in Iran. There are some studies on the assessment of normal language development as well as developmental language impairments in Iranian children. Nilipour et al. (58) compared quality of speech and the information-processing rate in Persian speaking children with specific language impairment (SLI). The results indicated that developmental language impairments of SLI children could be observed at different levels of language. Moreover, the slow rate of information processing has been reported as a feature of connected speech in SLI children, as compared with their age-matched peers (59). Nilipour et al. (59) also developed object and action naming battery in healthy children. The results indicated a significant noun advantage with regard to accuracy and naming latencies. The results also revealed that transitive verbs were named more accurately than intransitive ones in Persian speaking children. In another study, Nematzadeh et al. (60) published basic common concepts to be used in primary school text books developed in Persian. The results of these clinical linguistic studies can give us the basic clue and materials to develop language tasks for presurgical mapping in Iranian children. 

It is not still clear which language and memory paradigms produce optimal activation for children and how these should be quantified in a statistically robust manner (61). However, cultural and linguistic adaptation of presurgical tasks normed on a healthy aged-matched control group can give us a clue for clinical sensitivity of presurgical tasks in pediatric clinical settings.

**In Conclusion, **Precise presurgical language mapping as a non-invasive method has been proven to be helpful for children with intractable epilepsy to preserve their language abilities after surgery. Moreover, it has been indicated that epilepsy may affect normal language development and laterality in some children. Since possible language impairments can affect social interaction and academic achievements in epileptic children, it is highly essential to know how language is processed and represented in children with epilepsy and possible language disorders in epileptic children in both presurgical and postsurgical stages. Further, as the results of studies in clinical settings on pediatric epilepsy in other cultural settings indicated, it is not still clear which language and memory paradigms produce optimal activation for children and how these should be quantified in a statistically robust manner (61). 

Linguistic and culturally standardized presurgical language mapping paradigms are recommended for Persian speaking children with intractable epilepsy to preserve their language abilities after surgery. Finally, the results of fMRI language mapping as a non-invasive method based on culturally and linguistically standardized paradigms can help us to specify the laterality index and regions of interests to promote quality of life in the affected children, even in the post-surgery stage.

## Authors contribution

Mahdieh Karami: prepared the first draft of the paper 

Reza Nilipour, was the corresponding author of the manuscript and revised the article 

Majid Barekatain: Reviewed the related literature and contributed to the discussion 

William D. Gaillard: supported the preparation and writing of the manuscript, made substantial contribution in developing and eviewing the related literature and discussion.
